# Optical transparency and label-free vessel imaging of zebrafish larvae in shortwave infrared range as a tool for prolonged studying of cardiovascular system development

**DOI:** 10.1038/s41598-022-25386-w

**Published:** 2022-12-03

**Authors:** Mikhail Volkov, Alexander Machikhin, Valeriya Bukova, Demid Khokhlov, Alexander Burlakov, Viacheslav Krylov

**Affiliations:** 1 Scientific and Technological Center of Unique Instrumentation, Russian Academy of Sciences, Moscow, Russia 117342; 2 ITMO University, Saint Petersburg, Russia 197101; 3 Bauman Moscow State Technical University, Moscow, Russia 105005; 4 Lomonosov Moscow State University, Moscow, Russia 119991; 5 Papanin Institute for Biology of Inland Waters, Russian Academy of Sciences, Borok, Russia 152742

**Keywords:** Optical imaging, Animal physiology

## Abstract

Optical techniques are utilized for the non-invasive analysis of the zebrafish cardiovascular system at early developmental stages. Being based mainly on conventional optical microscopy components and image sensors, the wavelength range of the collected and analyzed light is not out of the scope of 400–900 nm. In this paper, we compared the non-invasive optical approaches utilizing visible and near infrared range (VISNIR) 400–1000 and the shortwave infrared range (SWIR) 900–1700 nm. The transmittance spectra of zebrafish tissues were measured in these wavelength ranges, then vessel maps, heart rates, and blood flow velocities were calculated from data in VISNIR and SWIR. An increased pigment pattern transparency was registered in SWIR, while the heart and vessel detection quality in this range is not inferior to VISNIR. Obtained results indicate an increased efficiency of SWIR imaging for monitoring heart function and hemodynamic analysis of zebrafish embryos and larvae and suggest a prolonged registration period in this range compared to other optical techniques that are limited by pigment pattern development.

## Introduction

Zebrafish (*Danio rerio*) is a small cyprinid fish. Powerful genetic tools, small body size, many offspring, and relative ease of laboratory manipulation make zebrafish one of the most common model organisms for studying various processes in vertebrates, including human diseases^1–3^. Last decades *D. rerio* have been actively used for modeling cardiovascular pathologies due to the presence of human disease genes in the genome^4^ and optical transparency of the embryos^5^. The zebrafish heart contains the most specialized structures and signaling pathways intrinsic to mammals and humans^6–8^. The basic vascular anatomy of the developing *D. rerio* also shows strong similarity to that of other vertebrates^9^. Mentioned advantages suggest that zebrafish models will keep their relevance in studying cardiovascular diseases in the future^10^.

Optical approaches are the most common for non-invasive studying of the zebrafish cardiovascular system at early developmental stages. These techniques are utilized in the zebrafish model as a tool for examining congenital heart defects^11^, cardiomyopathy^12–14^, as well as the effects of various influences on the cardiovascular system functioning^15–17^. Moreover, transparent zebrafish embryos can be used for studying cardiovascular phenotypes, which would be lethal in humans and other mammals, as they obtain oxygen by passive diffusion from water for the first several days of development^8^. Optical approaches are also applied to studying the impact of blood flow on the arrest and the extravasation of circulating tumor cells in zebrafish embryos^18^. It opens prospects for evaluating antitumor drug efficacy via studying the relationship between hemodynamics in the zebrafish model and cancer cell migration ability^2^.

Nowadays, optical techniques deliver outstanding imaging capabilities. Optical coherence tomography enables micrometer-resolution visualization of the internal zebrafish anatomy^19^. It becomes a reliable tool for identifying and studying specific tissues when coupled with photoacoustic^20^, angiographic^21^, or other imaging modalities. Confocal^22^ and light sheet^23^ microscopy provide a more detailed structure of zebrafish’s heart and vessels but require long-term scanning and blood cells labeling. Fluorescence of the dyes allows accurate high-contrast cardiovascular imaging^24^. Without them, extensive image segmentation and analysis algorithms are necessary^25^. Label-free and cost-effective optical approaches to heartbeat measurements and vessel mapping are mainly based on time-lapse analysis^26,27^ which means tracking the temporal variations related to blood pulsations in bright-field microscopic images.

Regardless of the physical principle, a significant restriction of optical techniques in visible light is that embryos lose their transparency due to pigment pattern formation. In wild-type zebrafish, this process commences at the prim-5 stage of embryonic development, approximately 24 h post fertilization ^28^. Then a few metamorphic melanophores scatter over the dorsal and ventral myotomes starting the transformation to the adult melanophore pattern in larvae^5^. It limits long-term experimental plans that utilize optical approaches by 1–2 weeks post-fertilization. Efforts have been made to develop techniques that keep the transparency of zebrafish embryos for a longer period^29^. Using 1-phenyl-2-thiourea that blocks the formation of melanophores due to the reduction of tyrosinase activity is proposed for this purpose^30^. Transparent zebrafish mutants as *TraNac* (*tra *^*b6/b6*^*; nac *^*w2/w2*^), *casper* (*roy *^*a9/a9*^*; nac *^*w2/w2*^), and *crystal* (*alb *^*b4/b4*^; *nac *^*w2/w2*^; *roy *^*a9/a9*^) are available^31–33^. However, genomic interventions^34^ or the application of chemical agents^35^ can affect experimentation results. Moreover, experimental plans may suggest utilizing wild-type zebrafish exclusively. Thus, the development of optical approaches allowing to ignore melanocytes is highly relevant for the zebrafish embryos model and fish embryos in general.

Optical techniques in SWIR (900–1700 nm) can be one of the ways to overcome this issue. Light in this range has advantageous properties such as reduced scattering and a higher penetration depth in biological tissues^36^. The availability of SWIR cameras enables multiple high sensitivity imaging modalities. In this paper, we demonstrate experimentally that the optical transparency of zebrafish tissues in SWIR is higher than in VISNIR that makes typical cardiovascular imaging observations much more effective.

## Materials and methods

### Specimen preparation

Wild-type *D. rerio* (AB strain) were obtained from the commercial distributor and maintained in the Laboratory of Physiology and Toxicology (Papanin Institute for Biology of Inland Waters, Russian Academy of Sciences). We kept zebrafish larvae in glass aquaria at 26 °C in a 12-h light–dark cycle and fed them with Artemia flakes. We put a selected individual into a 35 mm dish filled with water for each experiment and moved it back after image acquisition. We applied tricaine methanesulfonate (TMS, MS-222) solution at a concentration of 0.168 mg/mL within 1 min at 26 °C for anesthesia. After the experiments, the development, survival rate, morphology, and vital activity in the processed larvae did not differ significantly from those of not subjected specimens.

All methods were carried out in accordance with relevant guidelines and regulations. The study was approved by the Institutional Animal Care and Use Committee at the Papanin Institute for Biology of Inland Waters (protocol 6, date of approval: 25 February 2022 https://ibiw.ru/index.php?p=downloads&id=46958). In addition, the study was carried out in accordance with ARRIVE guidelines.

### Experimental setup

For image acquisition, we put the dish on a precise XYZ translation stage of transmitted light microscope BW Optics MF608. It has a 100 W halogen lamp equipped with continuous intensity adjustment and powered from the stabilized DC power supply. This light source has a wide irradiance spectrum that covers both VISNIR and SWIR. Adjustable lenses (collector and condenser) and diaphragms (field and aperture) of the microscope provide an even bright-field Kohler illumination of the sample. The imaging system consists of a microscopic objective (4 × NA 0.1), tube lens, adapter lens, and camera. Objective magnification may be adjusted depending on the task. It should be high enough to resolve single vessels and is a compromise of spatial resolution, depth of view, and data collection time. For a detailed analysis of a particular organ, one can install a 10 × or 20 × objective but make sure that the depth of focus is still acceptable (see Table 1). When a whole-body vessel image is required, it is necessary to scan the specimen by moving the translation stage with overlap between the fields of view at adjacent positions. The higher the resolution required, the more images and time we need to calculate vessel maps and stitch the whole-body panorama.

**Table 1:** Parameters of the experimental setup.

Spectral range	Pixel pitch	Image resolution	Microscopic objective	Adapter	Field of view	Depth of focus (μm)
VISNIR	5.5 × 5.5 μm^2^	1200 × 1200	4 ×	1 ×	1.86 × 1.86 mm^2^	10
			10 ×		0.6 × 0.6 mm^2^	5
			20 ×		0.24 × 0.24 mm^2^	2.5
SWIR	15 × 15 μm^2^	640 × 512	4 ×	0.5 ×	2.15 × 1.7 mm^2^	17
			10 ×		0.67 × 0.55 mm^2^	7
			20 ×		0.27 × 0.22 mm^2^	3.5

For VISNIR image acquisition, we had a monochrome CMOS camera IDS uEye UI-3360CP-NIR-GL Rev.2 (2048 × 1088 pixels, 5.5 × 5.5 μm^2^ pixel pitch). For SWIR imaging, we installed InGaAs camera Allied Vision Goldeye G-032 TEC2 (640 × 512 pixels, 15 × 15 μm^2^ pixel pitch). To compensate for the difference in the formats of these sensors and achieve closer values of optical magnification in VISNIR and SWIR, we applied 0.5 × and 1 × adapters, correspondingly.

Additionally, we inserted narrow band (FWHM 10 nm) filters with central wavelengths from 450 to 1600 nm with a 50 nm step into the microscope illumination system for spectral transmission measurements.

Before the experiments, we made sure that the optical transmission of microscope components was high enough in SWIR and carried out geometrical calibration of the setup. For this purpose, we acquired images of the grid distortion target and measured residual image distortion and illumination non-uniformity.

A series of N = 3000 images in VISNIR (12 bit, 1200 × 1200) and SWIR (14 bit, 640 × 512) at a 50 Hz frame rate within 60 s were collected in each experiment.

### Data processing

We applied a well-proven image processing pipeline described earlier^27^ to detect and quantify cardiac activity. First, we eliminated non-uniformity in illumination by subtracting low frequency components from all images and normalizing them. Second, we implemented precise pixel-to-pixel image stabilization necessary to compensate for relative twists and turns between the specimen’s parts. Third, we analyzed temporal intensity variations in each pixel of the well-matched image stack and detected pixels in which these signals have periodic shapes and relate to cardiac activity. Fourth, we obtained vessel images, calculated heartbeat, and measured blood flow velocity.

### Experimental protocol

**Figure 1:**
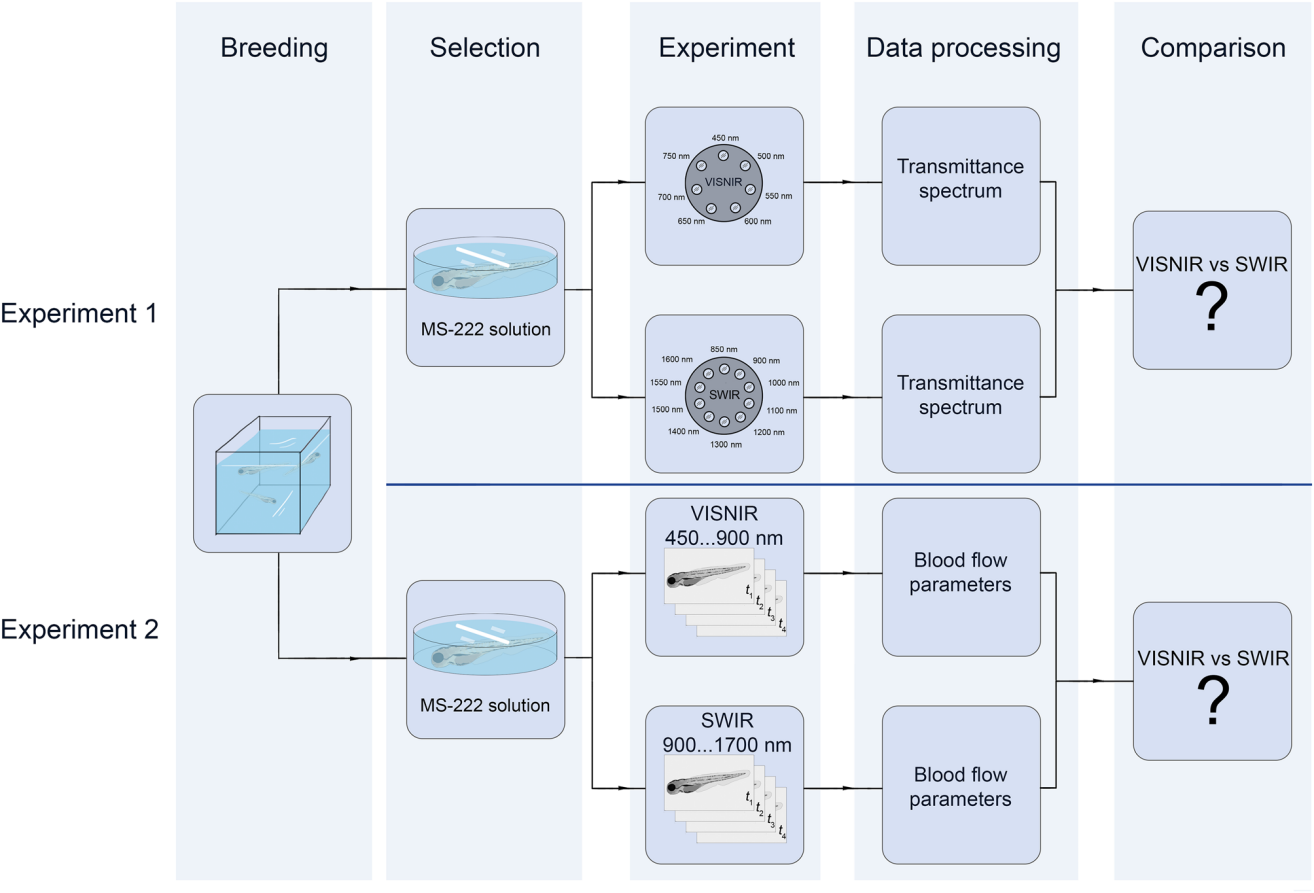
Experimental protocol. It consists of two sequential stages. In experiment 1, we measured the optical transparency of zebrafish tissues in the 450–1700 nm spectral range. In experiment 2, we calculated the parameters of the cardiovascular system in VISNIR and SWIR.

The study consisted of two stages (Fig. 1). The first one aimed to measure the spectral properties of zebrafish tissues and prove the potential advantages of SWIR over VISNIR in terms of optical transmittance. In the second stage, we compared the quantitative characterization of the cardiovascular system in VISNIR and SWIR by calculating vessel maps, heart rates, and blood flow velocities.

## Results

### Optical transparency measurements

**Figure 2:**
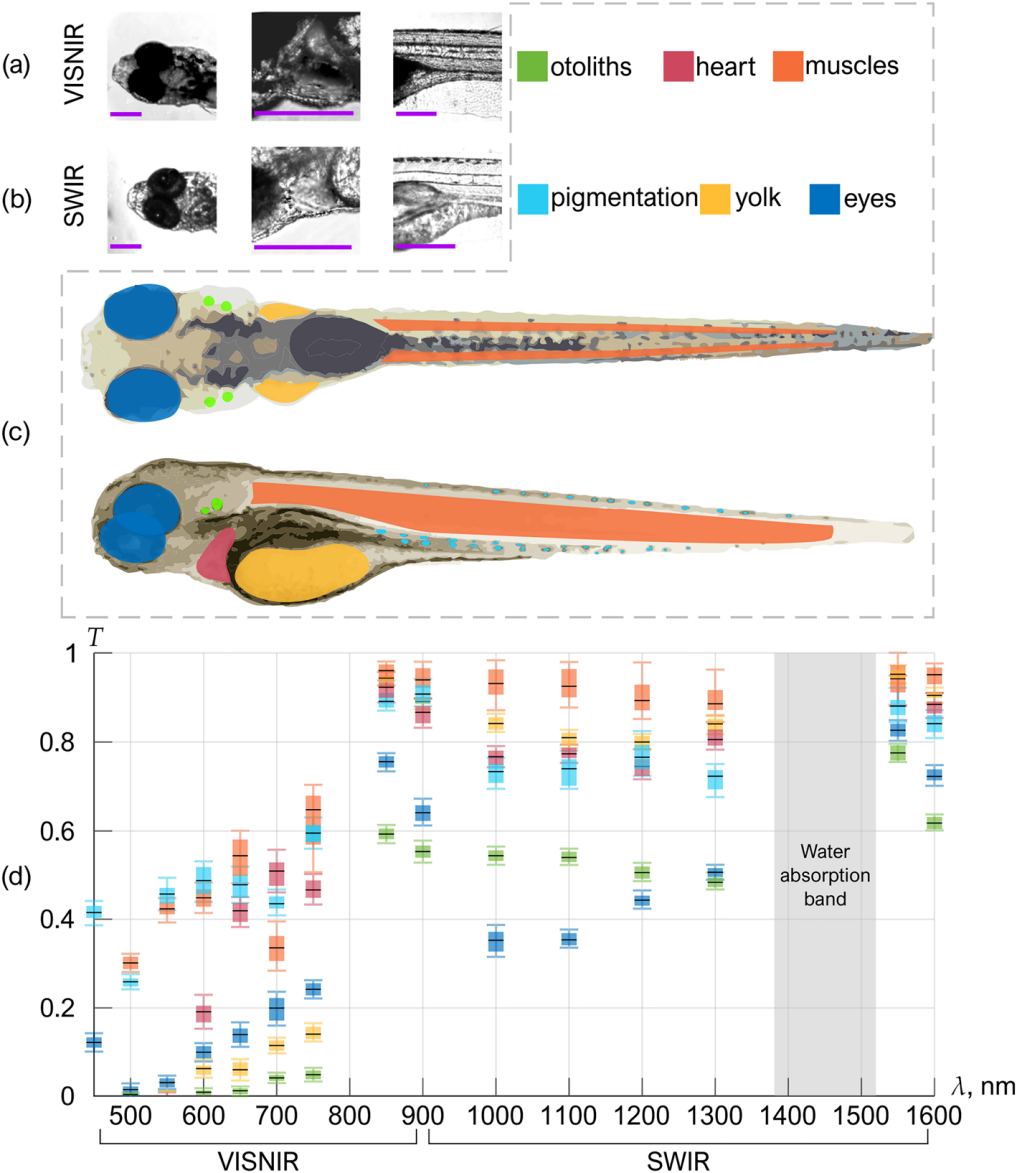
Examples of VISNIR (**a**) and SWIR (**b**) images, top and side views of the areas selected for spectral measurements (**c**), and calculated transmission (**d**) of zebrafish organs. Scale bar: 0.5 mm.

Figures 2a and 2b show VISNIR (400–900 nm) and SWIR (900–1700 nm) images of the eye, yolk, muscles, heart, and otoliths – organs that have different structures and properties^37^. We manually selected regions related to them (color-shaded areas in Fig. 2c) and marked the pigment pattern to see if its weak transparency in VISNIR differs in SWIR.

By installing the narrow-band filters into the microscope illumination system, we acquired a series of 15 spectral images of anesthetized zebrafish in the wavelength range 450–1600 nm, calculated wavelength dependences of the averaged intensity in selected organs *I*(*λ*_*i*_) (*i* = 1,2,..,15) and specimen-free background *B*(*λ*_*i*_), and evaluated transmission spectra as *T*(*λ*_*i*_) = *I*(*λ*_*i*_) /* B*(*λ*_*i*_). Figure 2d demonstrates the box plots *T*(*λ*_*i*_) averaged over 5 species. Due to the high water absorbance around 1450 nm shown by the grey vertical stripe in Fig. 2d, we consider the values of *T*(*λ*_*i*_) in this wavelength band to be unreliable and, therefore, skip it. Data shown in Fig. 2d clearly indicates 1.5–2.5 times increased optical transmission *T*(*λ*_*i*_) of all zebrafish organs in SWIR. Muscles, yolk, heart, and pigmented skin are consistently 70–90% transparent in the whole range of 850–1600 nm.

### Cardiovascular study

**Figure 3:**
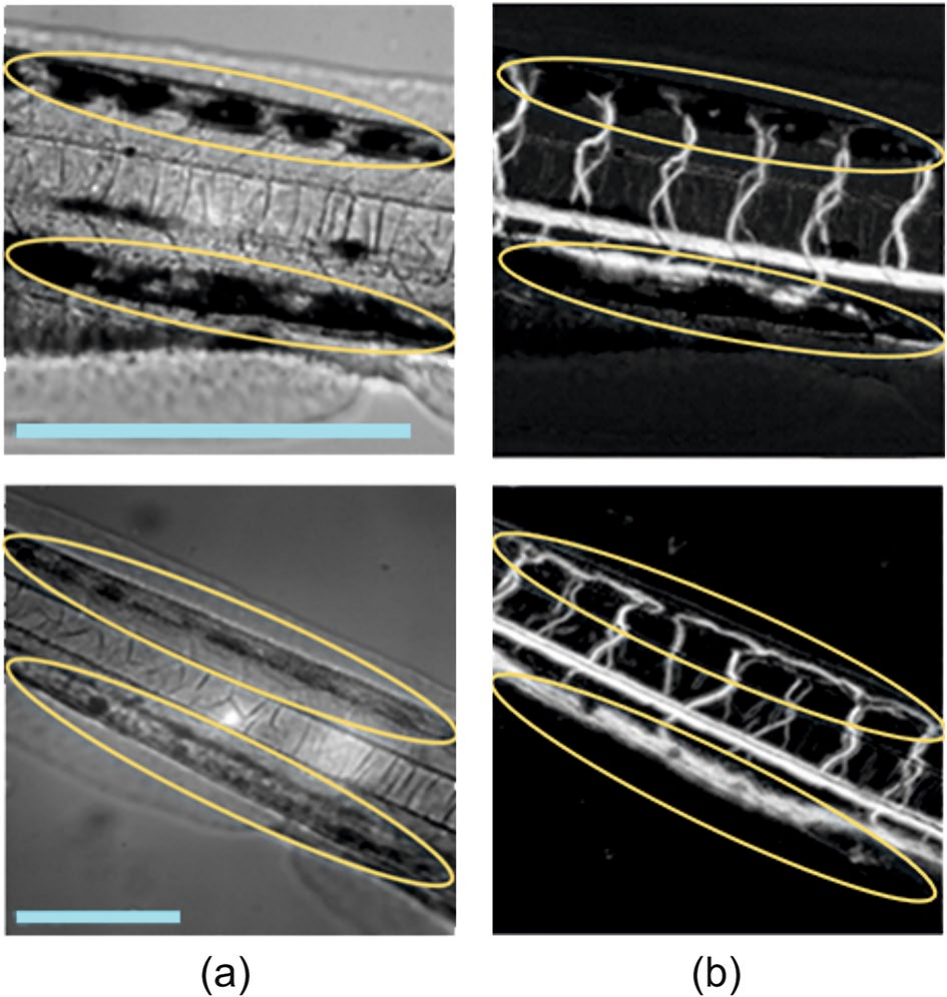
Example of acquired raw (**a**) and calculated vessel (**b**) images in VISNIR (upper row) and SWIR (lower row). Scale bar: 0.5 mm. Areas marked by yellow indicate the same parts of *vena cardinalis* and *arteria segmentalis* partly covered by pigment pattern.

*Vessel imaging.* Figure 3 illustrates typical VISNIR and SWIR images of zebrafish bodies. Once the images of the raw series are matched, videocapillaroscopy processing leads to calculation of blood flow maps, i.e. two-dimensional distributions of intensity variations and vessel images via highlighting the pixels in which intensity variations correspond to cardiac activity^27^. The detection of vessels from VISNIR images in the areas of strong pigmentation is barely possible. In the individual shown in Fig. 3, the pigment pattern marked with yellow covers a large part of *vena cardinalis* and partially *arteria segmentalis*. It immediately results in the absence of these vessels in the reconstructed vessel image. The pigment is transparent and, therefore, almost does not affect the blood flow detection capability of the image processing algorithm in SWIR. The vessel map reconstructed from the SWIR image series demonstrates a much more detailed structure of vessels including the ones hidden by pigmentation patterns.

**Figure 4:**
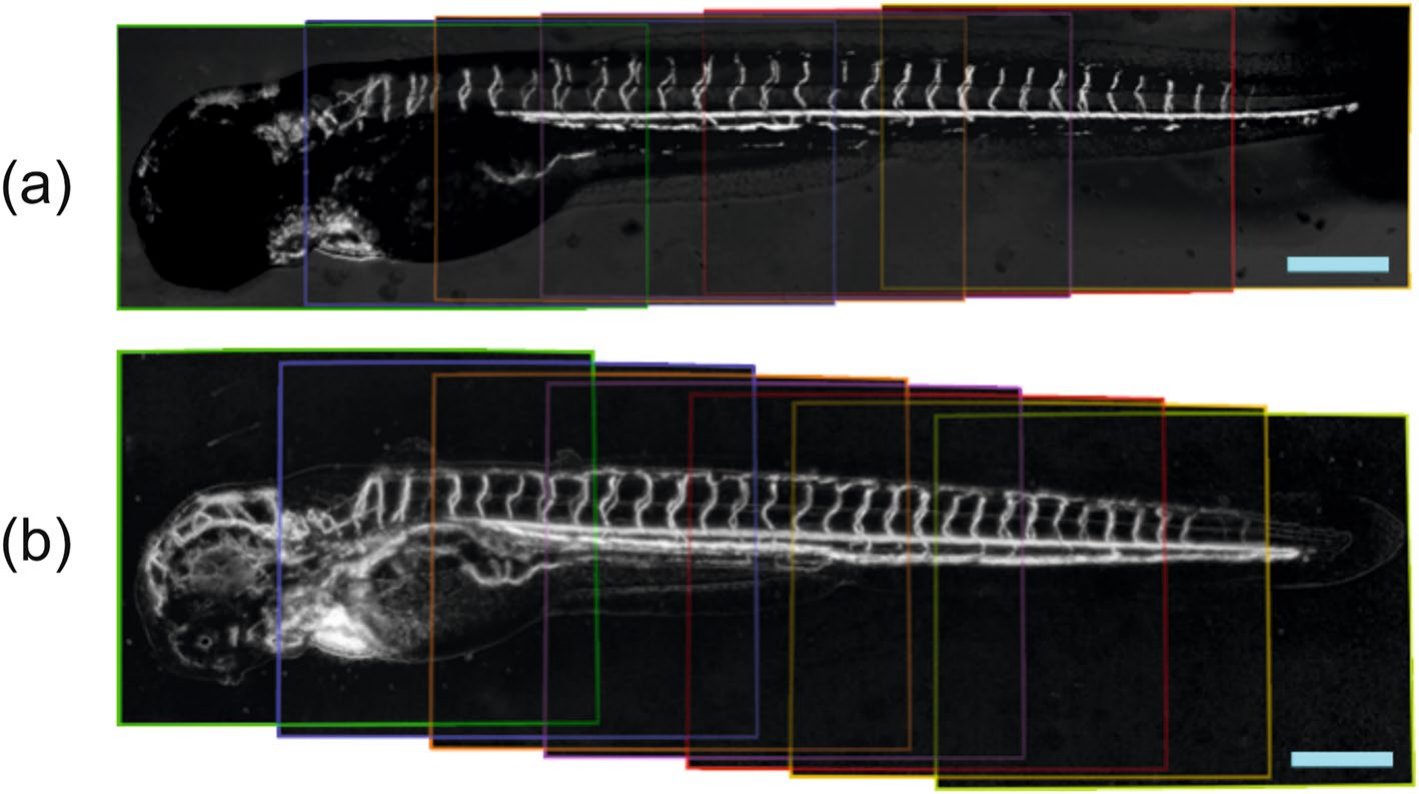
Panoramic vessel images in VISNIR (**a**) and SWIR (**b**). Scale bar: 0.5 mm. Color frames demonstrate the areas of the images from the initial stack.

We collected 8 image stacks with 20–30% overlap, calculated vessel maps, and stitched them into seamless panoramas to obtain high-resolution vessel images of the whole animal in VISNIR and SWIR (Fig. 4). In the SWIR panorama (Fig. 4b), one may see multiple vessels in the head, body, and tail which are absent in VISNIR (Fig. 4a). Again, due to transparency in SWIR, pigmentation all over the fish almost does not affect optical measurements.

**Figure 5:**
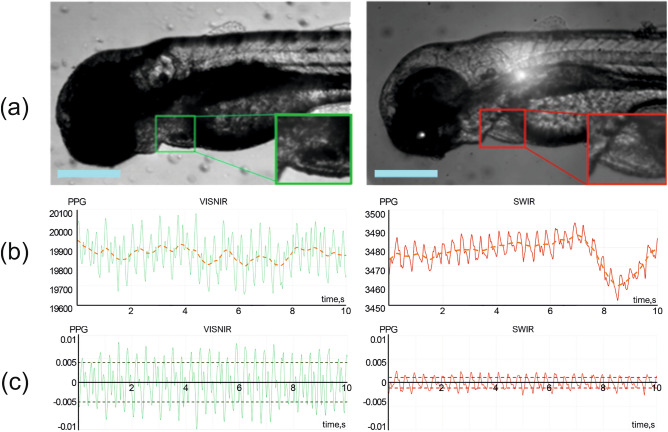
Raw images (**a**), initial (**b**), and normalized (**c**) PPG signals in the selected heart area in VISNIR (left) and SWIR (right). Scale bar: 0.5 mm. Dashed lines indicate the averaged PPG signal (**b**) and its root mean square values (**c**).

**Figure 6:**
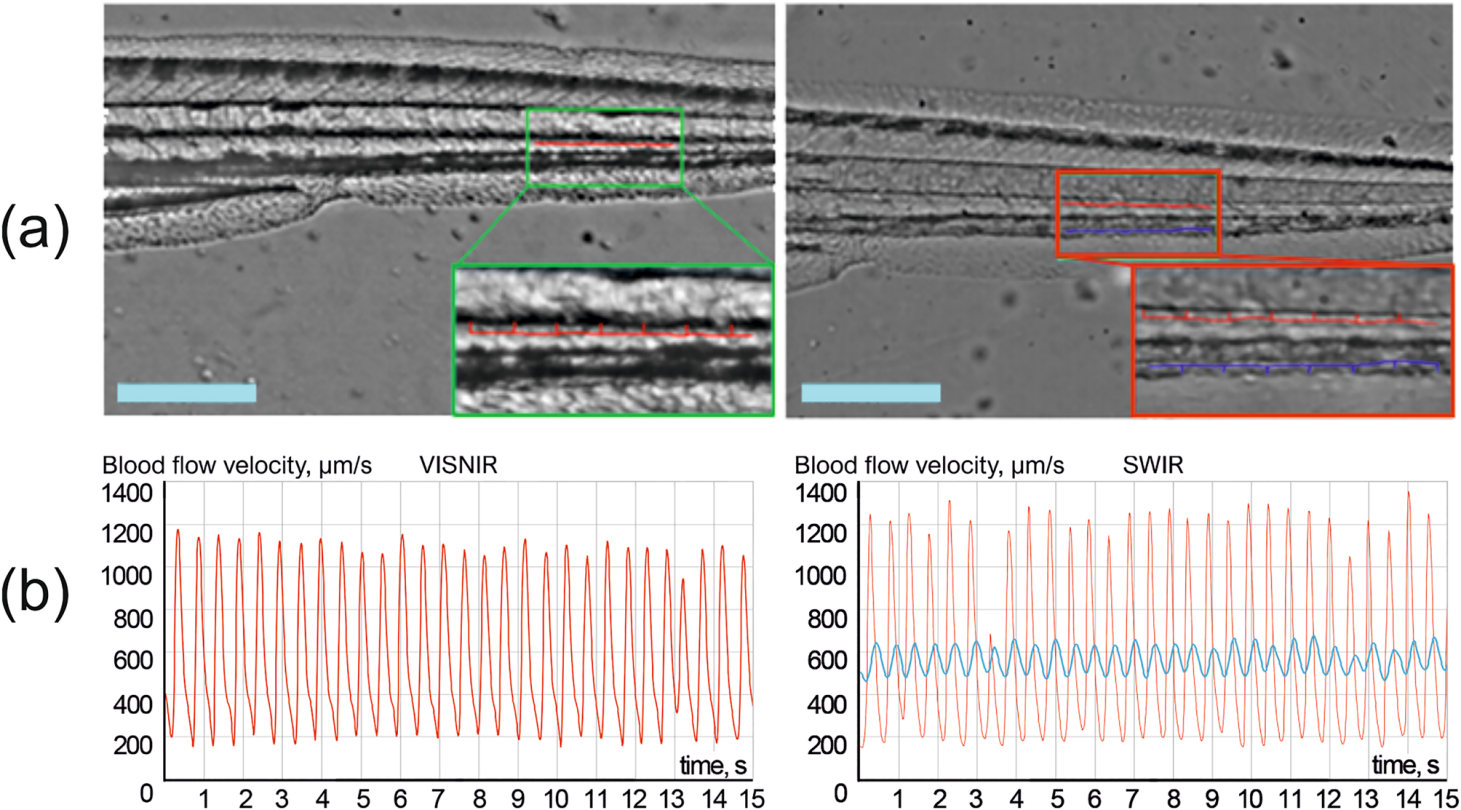
Raw images (**a**) and temporal dependences of blood flow velocity (**b**) in *aorta dorsalis* (red) and *vena cardinalis* (blue): VISNIR (left) and SWIR (right). Scale bar: 0.5 mm. Blood flow velocity in *vena cardinalis* was calculated only in SWIR due to the opaqueness of pigmentation that covers this vessel in VISNIR.

*Heart rate.* Heart rate is a primary indicator of cardiac function and overall health. Its measurement is a mandatory and well-established procedure performed by multiple optical techniques^38–42^. One of the most straightforward is photoplethysmography (PPG), i.e. tracking volumetric variations of blood circulation. We implemented this approach for heartbeat measurement in VISNIR and SWIR. We selected the heart area (highlighted with a color frame in Fig. 5a) and calculated the temporal dependency of its average intensity for this purpose (Fig. 5b). After subtracting low-frequency components, we obtained normalized PPG signals (Fig. 5c) that may be analyzed and compared. Our experiments show that both VISNIR and SWIR imaging provide clear and stable periodic PPG signals. Though the heartbeat signal in VISNIR has 4–5 times higher amplitude and a more detailed shape, both signals suit well for heart rate measurements. Fourier analysis allows calculating the dominating frequencies, i.e. periods of these signals. In the example shown in Fig. 6, the heart rates measured in VISNIR and SWIR are 161 bpm and 164 bpm correspondingly. These values are close to the data obtained in previous studies^22,43^.

*Blood flow velocity.* Another parameter crucial for quantitative characterization of cardiac function and understanding of cardiovascular hemodynamics is blood flow velocity^22,44^. Figure 6a shows raw images of a zebrafish body acquired in VISNIR and SWIR. We manually selected the areas of *aorta dorsalis* (red) and *vena cardinalis* (blue) (see Fig. 6a and 6b), transformed their trajectories into straight lines by detecting central lines of these vessels and calculating normals to them in each point, and tracked relative shifts between the adjacent images^27^. Then, we may calculate blood flow velocity as a product of this shift and frame rate of 50 Hz. The pigmentation pattern almost completely covers *vena cardinalis* in VISNIR (see Fig. 6a). Thus, its detection and analysis are feasible only in SWIR. Figure 6b shows temporal dependences of blood flow velocity in *aorta dorsalis* and *vena cardinalis*. The phase shift between the signals in *aorta dorsalis* and *vena cardinalis*, their shapes, velocity values (VISNIR: 576 ± 450 μm/s, SWIR: 574 ± 510 μm/s and 564 ± 112 μm/s), and other features correspond well to the data obtained by alternative methods^22,44^. We may also extract heart rate from these signals by simple frequency analysis. It is 161 bpm in VISNIR and 164 bpm in SWIR in the example shown in Fig. 6. These values are consistent with that obtained from PPG signals in the heart area (Fig. 5c).

Table 2 summarizes the quantitative data related to the presented cardiovascular studies in VISNIR and SWIR for two zebrafish 4 dpf larvae. The signal-to-noise ratio (SNR) of PPG signal shows the reliability of cardiac activity measurements. The vessel density was defined as the proportion of the pixels related to vessels with detected blood flow over the total amount of pixels occupied by the specimen. Quantifications in Table 2 reveal the advantages of cardiovascular studies in SWIR over conventional VISNIR imaging. The processing of SWIR images is much more informative and reliable in terms of vessel mapping and blood flow velocity measurement due to the increased transparency of zebrafish organs and pigmentation.

**Table 2:** Quantitative cardiovascular data measured in VISNIR and SWIR.

Spectral range	Specimen	SNR of PPG, dB		Heartbeat, bpm	Vessel density			Blood flow velocity, μm/s	
		Heart	Vessels		Head	Body	Tail	*aorta dorsalis*	*vena cardinalis*
VISNIR	#1	9.42	10.18	161 ± 3.5	0.089	0.116	0.101	576 ± 450	Not detected
	#2	9.17	9.85	159 ± 4.6	0.081	0.104	0.099	519 ± 399	Not detected
SWIR	#1	3.39	3.44	164 ± 3.8	0.338	0.271	0.194	574 ± 510	564 ± 112
	#2	3.25	3.31	155 ± 5.5	0.352	0.299	0.197	521 ± 407	505 ± 407

## Discussion

Our evaluation of the VISNIR and SWIR imaging showed a significantly increased optical transmission for zebrafish tissues and greater transparency of pigment pattern in SWIR. At the same time, the quality of heart and vessels detection in the images obtained in this range is not inferior to VISNIR. PPG signals derived from SWIR images are consistent with the heart rhythm. In this way, relatively simple changes in technical implementation make it possible to extend the period for the non-invasive studying of the cardiovascular system in zebrafish larvae and juveniles by the optical methods up to the formation of definitive scales^5^. This technique reveals new prospects for utilizing zebrafish as models for development and disease via long-term experimental plans for studying congenital heart defects, cardiomyopathy, and the effects of various influences on the functioning of the cardiovascular system^11–17^. In addition, long-term experiments are preferred for studying the dynamics of metastasis via circulating tumor cells in the zebrafish bloodstream^2^. Extended registration of cardiovascular performance and angiography in such studies can be applicable for predicting the efficiency of personalized cancer therapy and exploring mechanisms that drive metastasis and responses to therapy.

Transparent zebrafish larvae are also widely used for the assessment of drug-induced cardiotoxicity^45^. This kind of toxicity is one of the most frequent reasons for drug withdrawals^46^. Harmful reactions identified with the zebrafish model before clinical trials reduce the cost of testing new drugs^45,47^. Optical approaches are often applied to evaluating the functioning of the *D. rerio* cardiovascular system for drug screening^47–49^. Revealed advantages of SWIR imaging can provide an evaluation of the chronic cardiotoxicity in zebrafish with the non-invasive technique. The prolonged SWIR imaging registration period will allow more accurate preclinical studies, including delayed effects of low concentrations of drugs and possible bioaccumulation of drugs. Moreover, long-term experimental plans are necessary to assess the pharmacodynamics of drugs. Thus, the approach described and tested in the present study can significantly expand the possibilities of *D. rerio* utility as a drug screening tool.

Scales cover the zebrafish with age, and an adult iridophore-xanthophore pigment pattern appears in addition to melanophores^5^. It can reduce the transparency of the adult zebrafish in the SWIR range. SWIR imaging is suitable for monitoring heart function and hemodynamic analysis in juvenile zebrafish and fry. Imaging of 10 dpf zebrafish in VISNIR and SWIR can be found in the Supplementary Video. The main advantage of SWIR imaging is the almost complete transparency of the melanophores. It allows extending the imaging period in wild-type zebrafish by 2–3 weeks compared to optical techniques and abandoning genetic or biochemical manipulations to increase tissue transparency. This approach can be used to build complex long-term experimental plans in the zebrafish model (for example, when studying the pharmacodynamics of drugs, delayed effects and bioaccumulation of drugs and xenobiotics, the dynamics of metastasis via circulating tumor cells in the bloodstream, etc*.*).

The zebrafish is by far the most in-demand fish in biomedical experiments today. A smaller but significant part of studies is carried out on medaka *Oryzias latipes*^50^. Nowadays, some other fish species among teleosts are explored for modeling human disease^51^. The early development of fish species is quite similar^37^, so the non-invasive optical approaches with SWIR imaging most likely can be implemented for studying the cardiovascular performance and angiography in most teleosts species at early development.

## Conclusion

Zebrafish tissues and their pigmentation pattern demonstrate increased transparency in SWIR in comparison to VISNIR. This finding expands the scope of optical techniques for cardiovascular research as monitoring cardiac activity and blood flow imaging. Even in the presence of a pigment, SWIR imaging allows non-invasive mapping and quantification of vessels located behind it. We believe that the results of this study may improve the efficiency of multiple optical techniques (photoplethysmography, optical coherence tomography, hyperspectral imaging, etc.) widely used for studying developmental dynamics and disorders in zebrafish.

## Supplementary Information

Supplementary Video 1.

Supplementary Video 2.

## Data Availability

The main data generated and analyzed during this study are included in this published article. The additional datasets generated and analyzed during the current study are available from the corresponding author on reasonable requests.
